# The SAX-3 Receptor Stimulates Axon Outgrowth and the Signal Sequence and Transmembrane Domain Are Critical for SAX-3 Membrane Localization in the PDE Neuron of *C. elegans*

**DOI:** 10.1371/journal.pone.0065658

**Published:** 2013-06-12

**Authors:** Jia Li, Pu Pu, Weidong Le

**Affiliations:** The Key Laboratory of Stem Cell Biology, Institute of Health Sciences, Shanghai Institutes for Biological Sciences, Chinese Academy of Sciences, Shanghai, China; National Institutes of Health (NIH), United States of America

## Abstract

SAX-3, a receptor for Slit in *C. elegans*, is well characterized for its function in axonal development. However, the mechanism that regulates the membrane localization of SAX-3 and the role of SAX-3 in axon outgrowth are still elusive. Here we show that SAX-3::GFP caused ectopic axon outgrowth, which could be suppressed by the loss-of-function mutation in *unc-73* (a guanine nucleotide exchange factor for small GTPases) and *unc-115* (an actin binding protein), suggesting that they might act downstream of SAX-3 in axon outgrowth. We also examined genes related to axon development for their possible involvement in the subcellular localization of SAX-3. We found the *unc-51* mutants appeared to accumulate SAX-3::GFP in the neuronal cell body of the posterior deirid (PDE) neuron, indicating that UNC-51 might play a role in SAX-3 membrane localization. Furthermore, we demonstrate that the N-terminal signal sequence and the transmembrane domain are essential for the subcellular localization of SAX-3 in the PDE neurons.

## Introduction

During the development of nervous system, an intriguing feature is that an individual neuron projects its axon precisely to its target and then makes functional synapse connection. The growth cone at the leading edge of an axon can sense attractive and repulsive ligands through different receptors to navigate along specific path. In the past twenty years, numerous axon guidance ligands and receptors have been identified, and most of them are conserved among species from worms to mammals [1]–[4].

In *C. elegans,* the main cues for axon development along the dorsal-ventral (D/V) axis are UNC-6 in the ventral nerve cord and SLT-1 in the dorsal nerve cord. UNC-6 is a homolog of Netrin, whereas SLT-1 is a homolog of Slit in vertebrates [5], [6]. UNC-6 acts through its receptors, UNC-40 (a homolog of DCC in vertebrate) and UNC-5 in axon guidance. Both of UNC-40 and UNC-5 are single transmembrane receptors that belong to an immunoglobulin super family [7], [8]. UNC-40 receptor is required for axon ventral guidance that are attracted by UNC-6, whereas both UNC-5 and UNC-40 are required for axon dorsal extension that are repelled by UNC-6 [9], [10]. SLT-1, which is secreted from the dorsal muscles, repels axons that express SAX-3 receptor toward the ventral nerve cord [11].

In *C. elegans*, *sax-3* encodes a single-pass transmembrane receptor that is an ortholog to *Drosophila* Robo. SAX-3 is transiently expressed in developing neurons during axon outgrowth [12]. Mutations in *sax-3* lead to axon abnormal midline crossing, long-range cell migration defects, ventral axon guidance failure, and nerve ring formation defects [12], [13]. A *C. elegans* Slit ortholog, SLT-1, is a ligand for SAX-3 in directing axon guidance decision [11]. Many studies that focus on the mechanisms that regulate neuronal development by SAX-3 have been published [14]–[18]. However, how SAX-3 is localized in neurons, and the genes or pathways that may affect the membrane localization of SAX-3 remain to be elucidated.

In this study, we found that a functional SAX-3::GFP, which could rescue axon guidance defect of *sax-3* mutant worms, stimulated ectopic axon formation in the PDE neurons. This effect was dependent on a guanine nucleotide exchange factor (GEF) UNC-73 and an actin binding protein UNC-115. The functionalities of the guidance receptors are probably regulated by their localizations [19]. We therefore examined the distribution of SAX-3::GFP in mutants with axon guidance defect to identify genes that might play an important role in SAX-3 membrane localization. We found that in the *unc-51* mutants or *unc-51RNAi* worms, approximately five percent of the PDE neurons contained abnormal SAX-3::GFP aggregates in the neuronal cell bodies, suggesting that UNC-51 might participate in the distribution of SAX-3 in the PDE neurons. To search for the signal that are important for SAX-3 membrane localization, we expressed different truncated SAX-3 receptors separately in the PDE neurons and observed that the N-terminal signal sequence and the transmembrane domain were essential for SAX-3 receptor membrane localization.

## Materials and Methods

### Molecular Biology

A 802 bp *dat-1* promoter was amplified by KOD-PLUS Neo DNA polymerase (YOBOBO) from the genomic DNA of wild-type N2 worms. The *dat-1* promoter was then inserted into the *BamH*I and *Not*I sites of pPD117.10 to generate pPD117.01-P*dat-1*::GFP. The full coding sequence of *sax-3* was amplified from wild-type cDNA, and was then subcloned into the *Not*I and *Age*I sites of pPD117.01-P*dat-1*::GFP to generate pPD117.01-P*dat-1*::SAX-3::GFP. The full length cDNA of *sax-3* with a TAATAA stop code was inserted into *Not*I and *Age*I sites of pPD117.01-P*dat-1*::GFP to generate pPD117.01-P*dat-1*::SAX-3. The truncated SAX-3 receptors were amplified and cloned in to the same sites of pPD117.01-P*dat-1*::GFP to generate the pPD117.01-P*dat-1*:: truncated SAX-3::GFP.

N-terminal single sequence of *sax-3*: 1–126 bp.

*sax-3* without the N-terminal single sequence: 127–3807 bp.

Extracellular domain of *sax-3*: 1–2625 bp.

Transmembrane domain *sax-3:* 2626–2688 bp.

Intracellular domain *sax-3*: 2689–3807 bp.

### Transformation of *C. elegans*

Extrachromosomal arrays were attained by injection into the germline of N2 worms using standard techniques [20]. Each sample of 50 ng/µl DNA and 5 ng/µl *myo-2*::mcherry was injected into the adult gonad. Two independent transgenic lines were generated for each construct. P*dat-1*::SAX-3::GFP and P*dat-1*::SAX-3 transgenes were integrated into genome to generate the stable transgenic lines by exposing the worms to 4,5′,8-trimethylpsoralen (Sigma-Aldrich) combined with UV light. Two independent stable lines for each variant were generated and the lines were outcrossed to N2 worms for six times. The main transgenic strains we generated in this study include: *ngEx*31(P*dat-1*::SAX-3::GFP), *ngIs15(*P*dat-1*::SAX-3::GFP*)*, *ngIs18*(P*dat-1*::SAX-3).

### Strains

The Bristol strain N2 was used as the standard wild-type worm. Experiments were performed at 20°C using standard *C. elegans* techniques [21]. The strains used in this paper are listed below:

Mutant strains:

LG I: unc-73(e936), unc-40(n324), unc-11(e47), and unc-101(m1).

LG II: *rrf-3(pk1426)* and *unc-104(e1265).*

LG III: *unc-116(e2310).*

LG IV: unc-5(e53), ced-10(n1993), and egIs1(Pdat-1::GFP).

LG V: unc-51(e369), unc-51(e1189), vab-8(e1017), and unc-34(e315).

LGX: unc-6(ev400), sax-3(ky123), slt-1(eh15), mig-2(mu28), unc-115(ky275), and *lqIs2(*P*osm-6::GFP).*

We crossed rrf-3(pk1426) into egIs1 and neIs15 to generate egIs1;rrf-3(pk1426) and ngIs15;rrf-3(pk1426) for all RNAi experiments.

### RNAi Experiments

Feeding RNAi was performed according to standard procedures [22] in *egIs1;rrf-3(pk1426)* and *neIs15;rrf-3(pk1426)* worms. For constructing *unc-51RNAi* vector, *unc-51* cDNA sequence corresponding to 1–1230 bp was cloned into the L4440 vector. s*ax-3, unc-73, unc-115, ced-10, mig-2, bec-1, atg-7, atg-18,* and *lgg-1* RNAi clones were made by the primers according to GenePairs primer sequences [22]. The empty vector L4440 was used as the control RNAi clone.

### Fluorescence Microscopy

Worms were immobilized in 5 mM sodium azide in M9 buffer on 2% agar pad slides. Images were taken by Leica TCS SP5 laser confocal scanning microscope. The PDE neuron was visualized by *egIs1(*P*dat-1::GFP)* or *lqIs2(*P*osm-6::GFP)* in different mutants. If the PDE axon failed to reach the ventral nerve cord or wandered beyond a 45-degree angle from a straight line ventrally from the cell body, it was considered to have ventral axon guidance defect [23]. A PDE neuron was scored as exhibiting ectopic axons if one or more extra axons were seen emanating from the normal axon or from the PDE cell body [24].

### Quantitative Real-time PCR and Primers

We performed real-time PCR to quantify the mRNA levels of genes. Real-time PCR amplifications were running on Applied Biosystems 7500 Real-Time PCR System in a mixture of SYBR Green Real-time PCR Master Mix (TOYOBO) and 0.4 mM of each primer in a final volume of 20 µl. The mRNA levels relative to the wild-type N2 worms were normalized to two endogenous reference genes (*act-1* and *ama-1*), and were calculated using the Applied Biosystems 7500 software V2.0.5. The primers used were listed as follows:

*ama-1* (forward primer: 5′-CGGTCAGAAAGGCTATCGAG-3′;

reverse primer: 5′-CCAACCTCCTGACGATTGAT-3′),

*act-1* (forward primer: 5′- GCTGGACGTGATCTTACTGATTACC -3′;

reverse primer: 5′- GTAGCAGAGCTTCTCCTTGATGTC -3′),

*bec-1* (forward primer: 5′- ACGAGCTTCATTCGCTGGAA -3′;

reverse primer: 5′- TTCGTGATGTTGTACGCCGA -3′),

*atg-18* (forward primer: 5′- CAGGAGCCGCAAGGAGTAAT -3′;

reverse primer: 5′- CGATTGGTTGCTTGCTTCGG -3′),

*atg-7* (forward primer: 5′- CCAAAAGCTGTGGGATGGGA -3′;

reverse primer: 5′- GCGTTCCAGCACCAAGAATG -3′),

*lgg-1* (forward primer: 5′- GCCGAAGGAGACAAGATCCG -3′;

reverse primer: 5′- GGTCCTGGTAGAGTTGTCCC -3′),

### Statistical Analysis

All values were shown as means ± SEM. A data analysis was performed using one-way ANOVA followed by Tukey’s post-hoc multiple comparisons or a *t*-test using the GraphPad Prism5 version 5.02 software.

## Results

### PDE Axon Guidance Defect in the *sax-3(ky123)* Mutant

**Figure 1:**
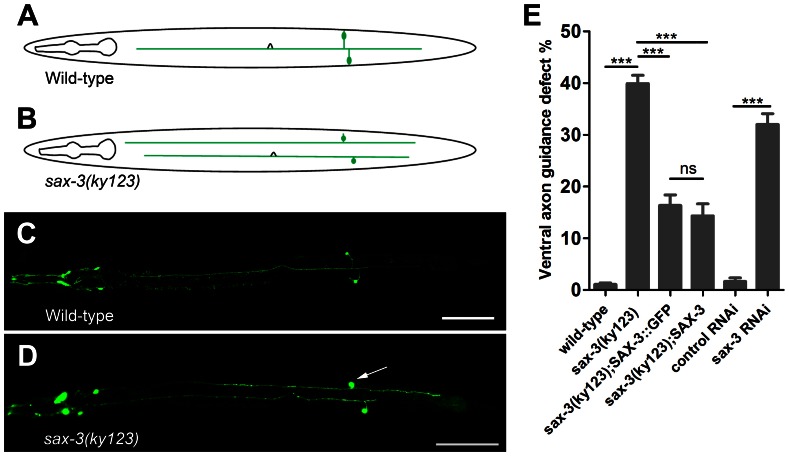
SAX-3 regulates axon guidance in PDE neuron. (A) and (B) Schematic diagrams of PDE neurons of a wild-type worm and a *sax-3(ky123)* mutant. (C) and (D) Confocal images of PDE neurons of a wild-type worm and a *sax-3(ky123)* mutant. The PDE neurons were visualized by *egIs1(*P*dat-1::GFP).* Arrow: a PDE neuron with ventral axon guidance defect. Scale bar: 100 µm. (E) Quantification of the PDE axon guidance defect in wild-type, *sax-3(ky123), sax-3(ky123);ngIs15(*P*dat-1*::SAX-3::GFP*)*, *sax-3(ky123);ngIs18*(P*dat-1*::SAX-3), and *sax-3RNAi* worms. At least 200 PDE neurons were examined and the results were averaged for each strain. ***P<0.001. ns: no significance. In all pictures, anterior is to the left. Error bars represent standard errors.

We analyzed the effect of *sax-3* on the axon development of the PDE neurons. The two PDE neurons, which locate at the post-deiridic region of *C. elegans*, are a bilateral pair of dopamine neurons that are born during the second larval stage. The PDE neurons can be visualized by GFP under the control of the *dat-1* promoter (P*dat-1*::GFP) or the *osm-6* promoter (P*osm-6*::GFP) [24], [25]. Each PDE neuron has an axon that extends straightly to the ventral nerve cord, where the axon bifurcates and extends both anteriorly and posteriorly (Figure 1A and 1C). We examined the PDE axon in the null *sax-3(ky123)* mutants, which lack the N-terminal signal sequence of the *sax-3* gene [12]. The axon of PDE neuron frequently failed to move ventrally to the ventral nerve cord (Figure 1B and 1D). Approximately 40% of the PDE axons exhibited ventral guidance defect in the *sax-3(ky123)* mutants (Figure 1E). To confirm the role of SAX-3 in PDE axon development, we knocked-down *sax-3* in *C. elegans* by RNAi. Because RNAi is generally ineffective in the neurons of *C. elegans*, RNAi experiments were performed using the *rrf-3(pk1426)* mutant worms, which is hypersensitive to RNAi [26] [19]. We found that the *rrf-3(pk1426);*P*dat-1::*GFP animals showed no detectable florescence of GFP in the PDE neurons after GFP RNAi treatment (Figure S1A and B). However, the wild-type P*dat-1*::GFP animals treated with GFP RNAi showed no decrease of green florescence in the PDE neurons (data not shown). Therefore, RNAi is effective in the *rrf-3(pk1426)* mutant worms. After knocking-down *sax-3* using the *rrf-3(pk1426);*P*dat-1::*GFP worms, we observed the similar ventral axon guidance defect in the PDE neurons (Figure 1E). To determine whether SAX-3 could rescue the PDE axon defect in the *sax-3(ky123)* mutant worms, we expressed SAX-3 or GFP-tagged SAX-3 (SAX-3::GFP) under the control of the *dat-1* promoter in the PDE neurons of *sax-3(ky123)* mutant animals. SAX-3::GFP has been proven to be functional and was used to determine the role of SAX-3 in axon development [27], [28]. To our expectation, we found that SAX-3 or SAX-3::GFP significantly reduced the percentage of the PDE axon guidance defect in the *sax-3(ky123)* mutant worms (Figure 1E), suggesting that SAX-3 has a cell-autonomous function in PDE axon guidance.

### SAX-3::GFP Induces Ectopic Axon Formation in PDE Neurons

**Figure 2:**
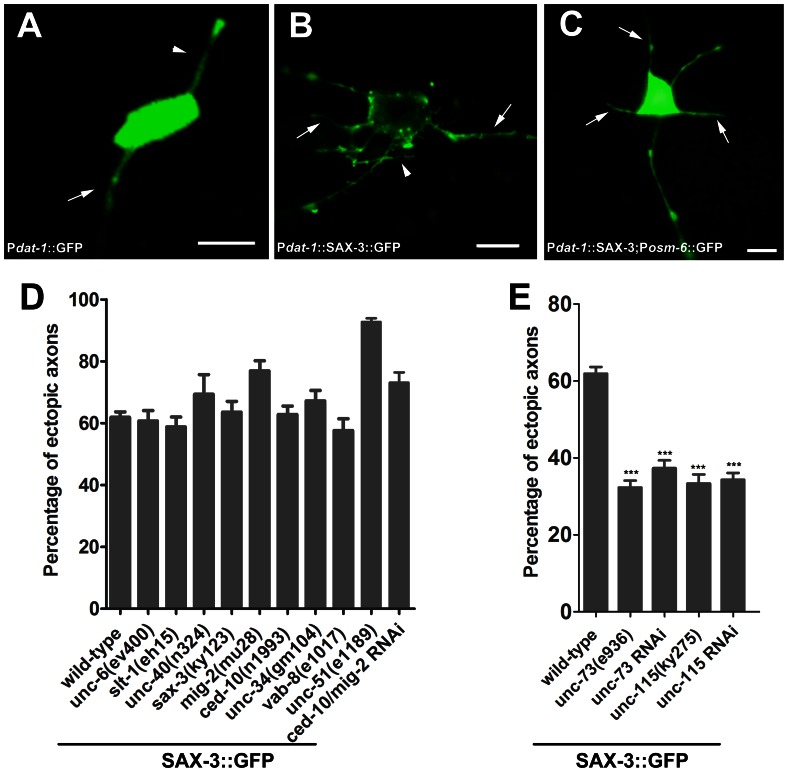
SAX-3::GFP causes ectopic axon formation which can be suppressed by *unc-73* and *unc-115*. (A) A wild-type PDE neuron with a short dorsal dendrite (arrowhead) and an axon (arrow). Scale bar: 5 µm. (B) SAX-3::GFP caused ectopic axon formation in the wild-type PDE neuron. The PDE neuron was visualized by *neIs15* (*dat-1*::SAX-3::GFP). Arrow: ectopic axons extending from the cell body. Arrowhead: ectopic axon emanating from the normal axon. Scale bar: 5 µm. (C) Over expression of SAX-3 induced ectopic axon formation in the PDE neuron. The PDE neuron was visualized by *lqIs2(*P*osm-6::GFP);ngIs18* (P*dat-1*::SAX-3). Arrowhead: ectopic axons. Scale bar: 5 µm. (D) *unc-6(ev400), slt-1(eh15), unc-40(n324), sax-3(ky123), mig-2(mu28), ced-10(n1993), unc-34(gm104),* and *unc-51(e1189)* failed to suppress ectopic axon formation in *neIs15* (*dat-1*::SAX-3::GFP). (E) *unc-73(e936), unc-73RNAi, unc-115(ky275)*, and *unc-115RNAi* partially suppressed the ectopic axon formation in *neIs15* (*dat-1*::SAX-3::GFP). RNAi was performed in *neIs15;rrf-3(pk1426)* animals. Asterisks denote statistically significant differences between the wild-type worms and the mutants. ***P<0.001. At least 200 PDE neurons were examined and the results were averaged for each strain in (D) and (E). Error bars represent standard errors.

**Figure 3:**
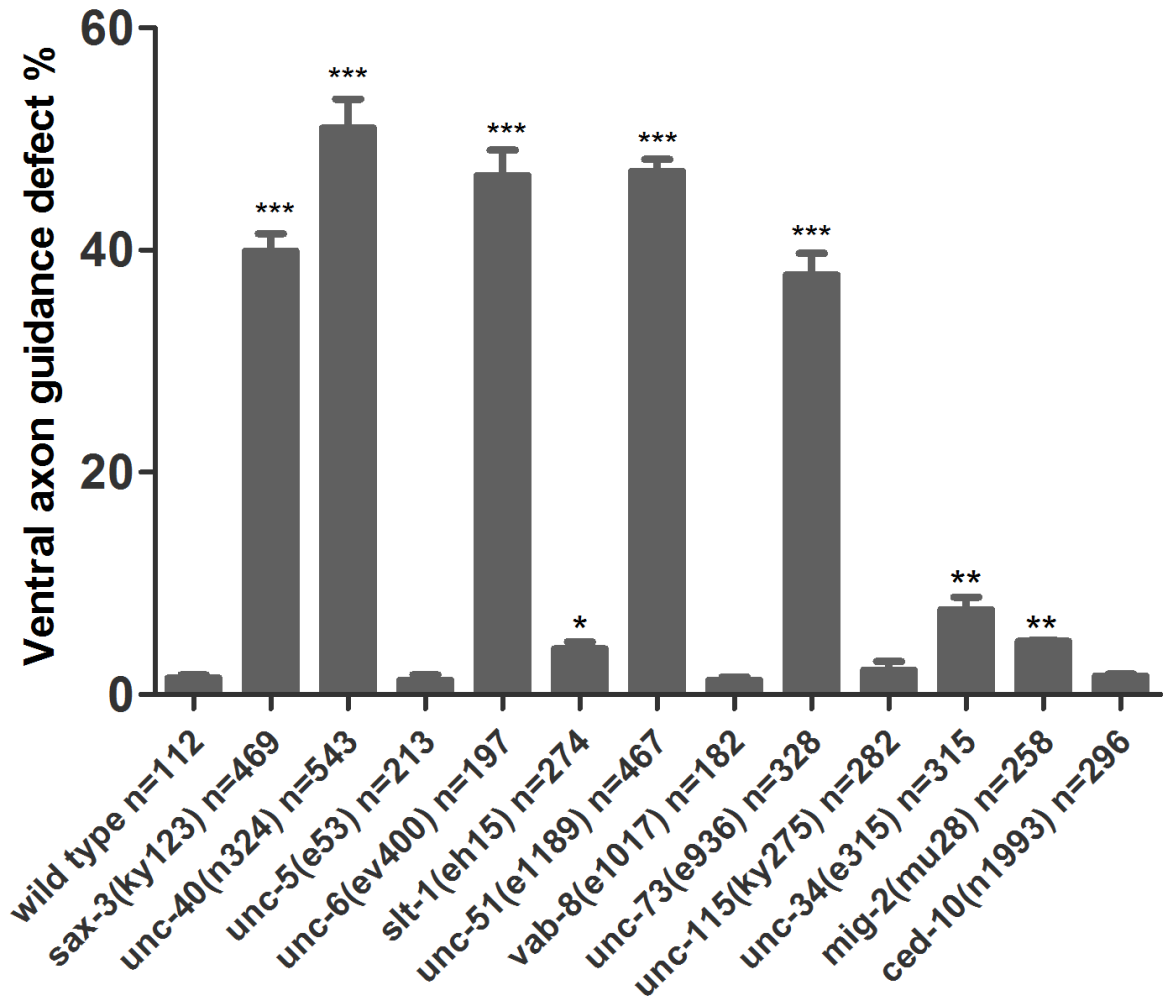
Quantification of PDE axon guidance defect in some *C. elegans* mutants. The PDE axon of *unc-5(e53)* and *ced-10(n1993)* mutants were examined by the *lqIs2(*P*osm-6::*GFP*),* other mutants were examined by the *egIs1(*P*dat-1::*GFP*)*. Error bars represent standard errors. Asterisks denote statistically significant difference between the wild-type worms and the mutants. ***P<0.001, **P<0.01, *P<0.05.

It has been reported that the functional MYR::UNC-40 stimulated ectopic axon outgrowth [29]. Therefore, we asked whether the functional SAX-3::GFP had the similar effect in axon outgrowth. We found that, compared with the wild-type PDE neurons, the PDE neurons that expressed SAX-3::GFP exhibited ectopic axon formation (ectopic axons emanated from the cell body or formed as branches from the main axon) (Figure 2A and 2B). We also overexpressed SAX-3 (without GFP tag) in the PDE neurons and observed similar phenotype (Figure 2C). We next tried to identify genes that might act in the SAX-3 pathway in axon outgrowth. We analyzed the genetic interaction between SAX-3::GFP and the known genes that participated in PDE axon development (Figure 3). Mutations in genes that act downstream of SAX-3 in axon outgrowth should suppress the ectopic axon formation caused by SAX-3::GFP. To do this, we crossed the SAX-3::GFP transgene to different mutants and analyzed the PDE axon morphology. First, we examined the genes in the Netrin/UNC-6 and the Slit-1/SLT-1 pathway. We found that loss of SLT-1 caused very weak ventral axon guidance defect (Figure 3), and *slt-1(eh15)* could not suppress the ectopic axon formation (Figure 2D). In addition, *unc-40(n324), unc-6(ev400),* and *sax-3(ky123)* failed to suppress SAX-3::GFP either (Figure 2D). Thus, SAX-3::GFP may act independently of endogenous SAX-3, SLT-1, UNC-40, and UNC-6 in axon outgrowth. Rac GTPases and actin-associated protein are involved in axon outgrowth and guidance, and they act as a molecular switch to control signal transduction pathway that links membrane receptors to the cytoskeleton signaling [30], [31]. Therefore, we next examined the Rac GTPases genes *ced-10* and *mig-2*. Although *ced-10(n1993)* could suppress excessive axons caused by MYR::UNC-40 [29], it could not suppress the ectopic axon formation by SAX-3::GFP (Figure 2D). MIG-2 also encodes a Rac GTPase and the null *mig-2(mu28)* mutant did not suppress SAX-3::GFP either (Figure 2D). It is known that *ced-10* and *mig-2* act redundantly in PDE axon development. Therefore, we performed double RNAi to know-down both of *ced-10* and *mig-2* in the SAX-3::GFP worms. However, ectopic axon formation could not been suppressed (Figure 2D). The loss-of-function mutant of *unc-34(gm104),* which could suppress MYR::UNC-40 [29], failed to suppress SAX-3::GFP (Figure 2D). UNC-73 encodes a Trio protein that acts as a GEF (Guanine nucleotide exchange factor) for Rac GTPase CED-10 and MIG-2 [32], [33]. We found that both *unc-73(e936)* and *unc-73RNAi* could partially suppress the ectopic axon formation by SAX-3::GFP (Figure 2E). These findings suggest that UNC-73 may regulate SAX-3::GFP induced axon outgrowth in a CED-10/MIG-2 independent manner. It is known that actin cytoskeleton arrangement plays an important role in axon outgrowth. UNC-115 is an actin-binding protein that controls lamellipodial and filopodial formation at the growth cone of axon, and is an effector of Rac signaling during axon guidance [23], [34]. It has been reported that UNC-115 acts downstream of the Netrin guidance receptor UNC-40 in axon outgrowth [29]. We observed that both of the *unc-115(ky275)* mutant and the *unc-115RNAi* treatment could partially suppressed the ectopic axon formation of the PDE neurons (Figure 2E), suggesting that actin rearrangement regulated by UNC-115 plays an important role in SAX-3::GFP induced axon outgrowth.

### UNC-51 may Participate in the Membrane Localization of SAX-3 in the PDE Neuron

**Figure 4:**
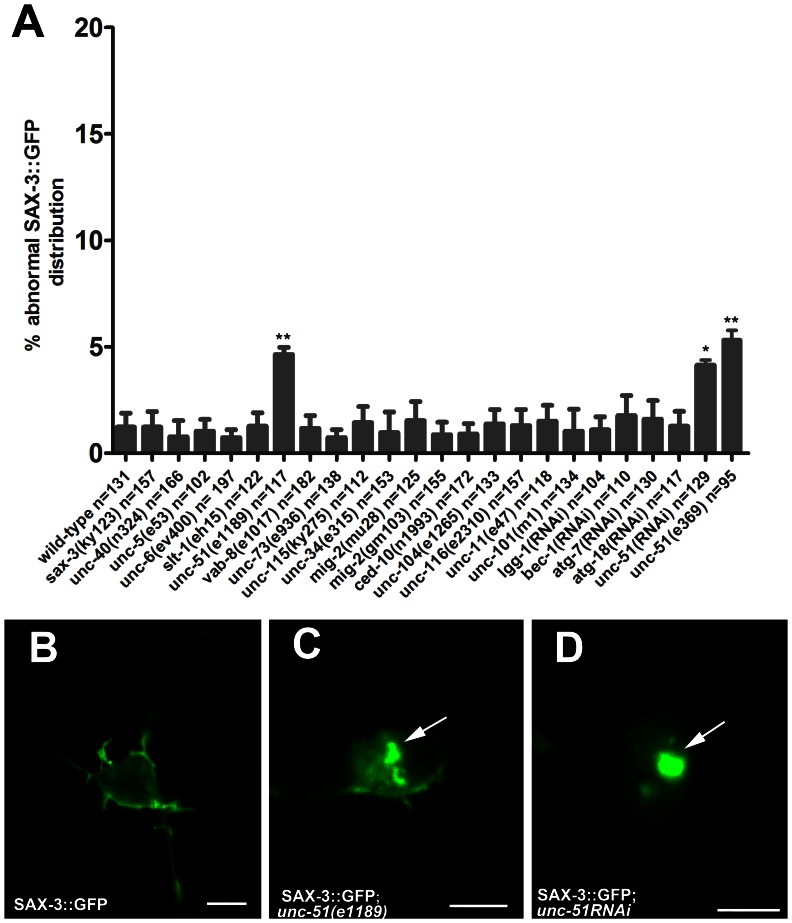
Localization of SAX-3::GFP in PDE neuron. (A) Quantitation of the abnormal SAX-3::GFP distribution in some mutants. Any SAX-3::GFP distribution that is different from (B) is regarded as abnormal. Asterisks denote statistically significant difference between the wild-type worms and the mutants. **P<0.01, *P<0.05. (B) In wild-type animals, SAX-3::GFP distributed mainly on cell membrane and axons, and was also evenly expressed in the cytoplasm. Scale bar: 5 µm. (C) *unc-51(e1189)* mutants showed altered SAX-3::GFP distribution in cytoplasm (with aggregates in cell body). Arrow: SAX-3::GFP aggregation. Scale bar: 5 µm. (D) SAX-3::GFP aggregates in the PDE cell body of the *unc-51* RNAi worm. Arrow: SAX-3::GFP aggregation. Scale bar: 5 µm. ***P<0.001. *ngEx*31(P*dat-1*::SAX-3::GFP) or *ngIs15*(P*dat-1*::SAX-3::GFP) was crossed to the mutants to examine the membrane localization of SAX-3::GFP. RNAi experiment was performed on *rrf-3(pk1426);ngIs15*(P*dat-1*::SAX-3::GFP) worms. Error bars represent standard errors.

To carry out their essential activities, guidance receptors must be localized correctly to cell membrane and axons. The regulation of guidance receptors traffic is important to the wiring of neuronal circuits [35]. Therefore, we next investigated the localization of SAX-3::GFP in the PDE neurons. It is reported that SAX-3::GFP is transiently expressed in most neurons [12]. We found that, in L4 stage wild-type worms, SAX-3::GFP was predominantly localized on the cell membrane and axons, and was also associated with small vesicles that were uniformly distributed in the cytoplasm (Figure 2B and 4B). To identify genes that participate in membrane localization of SAX-3, we firstly analyzed some known genes that were reported to affect guidance receptors traffic in *C. elegans*. VAB-8L is known to affect SAX-3::GFP expression level in the anterior lateral microtubule (ALM) neurons [18]. It is also known that *mig-2(gm103)* affects the membrane localization of UNC-40::GFP in the ALM neurons [28]. These led us to speculate that whether the membrane localization and traffic of SAX-3::GFP were regulated by these two genes. However, we didn’t observe any abnormal membrane localization of SAX-3::GFP in *vab-8(e1017)*, *mig-2(gm103)*, and *mig-2(mu28)* mutants (Figure 4A). Although *unc-73* and *unc-115* suppressed the ectopic axon formation caused by SAX-3::GFP, they did not affect SAX-3::GFP distribution in PDE neurons (Figure 4A). Next, we crossed SAX-3::GFP to several axon guidance mutants, including *unc-6, slt-1, unc-40, unc-34*, and *ced-10* mutants. The distribution of SAX-3::GFP in all these mutants were normal as observed in the wild-type worms (Figure 4A). We also determined the distribution of SAX-3::GFP in the kinesin mutants (*unc-104* and *unc-116*) and the clathrin adapter mutants (*unc-101* and *unc-11*). However, abnormal SAX-3::GFP distribution was not observed (Figure 4A).

UNC-51 is known for its unique role in vesicle trafficking of the receptor UNC-5 to the growth cone in dorsal axon guidance in *C. elegans* [19]. We found that although the distribution of SAX-3::GFP was normal in most PDE neurons, approximately five percent of the PDE neurons contained abnormal aggregates of SAX-3::GFP in *unc-51(e1189)* mutant worms, *unc-51(e369)* mutant worms, and *unc-51RNAi* worms (Figure 4A, 4C and 4D). Further, we performed CoIP assay to determine whether UNC-51 and SAX-3 have direct physical interaction. However, we did not detect any evidence of direct interaction between them. UNC-51 is a ortholog to yeast Atg1, which is required for the induction of autophagy [36]. Therefore, we asked whether it was an indirect consequence of broad effect caused by impaired autophagy. After knocking-down the autophagy-related genes *lgg-1, bec-1, atg-7*, and *atg-18* (Figure S1C), we did not observed any abnormal SAX-3::GFP subcellular distribution (Figure 4A), suggesting that these autophagic genes are not required for SAX-3 membrane localization in PDE neurons.

### The N-terminal Signal Sequence and the Transmembrane Domain of SAX-3 are Necessary for its Membrane Localization

**Figure 5:**
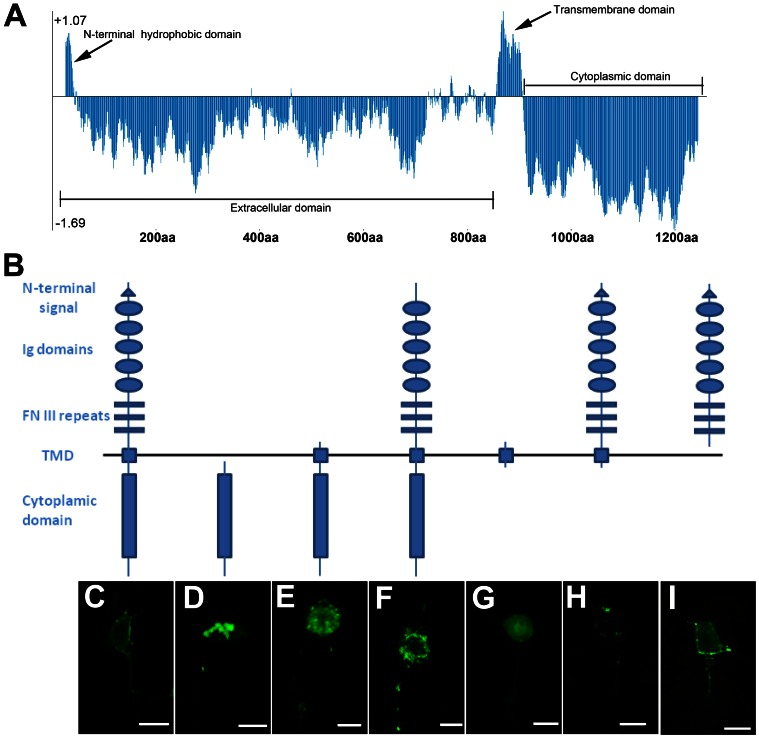
The localization of the truncated SAX-3 receptors in PDE neuron. (A) Hydropathicity analysis of the SAX-3 receptor. Arrows indicate the hydrophobic sequences on SAX-3. (B) SAX-3 is composed of three parts: the extracellular (Ex) domain (includes a N-terminal signal sequence, five immunoglobulin (Ig) domains, and three fibronectin type III (FN III) domains), the tramsmembrane (Tm) domain, and the cytoplamic (In) domain. (C) Full SAX-3::GFP in the PDE neuron. (D) In-SAX-3::GFP in the PDE neuron. (E) In-tm-SAX-3::GFP in the PDE neuron. (F) SAX-3::GFP without the N-terminal sequence in the PDE neuron. (G) Tm-SAX-3::GFP in the PDE neuron. (H) Ex-tm SAX-3::GFP in PDE neuron. (C)–(H) are shown in wild-type background. (I) Full length SAX-3::GFP in the null *sax-3(ky123)* background. (C)–(I) Scale bar: 5 µm.

To further explore the mechanism that controls SAX-3 membrane localization, we next tried to identify the localization signal that transported SAX-3 to cell membrane and axons. SAX-3 is a transmembrane receptor with an N-terminal hydrophobic sequence, five immunoglobulin domains, three fibronectin type III domains, a transmembrane hydrophobic domain, and a cytoplasmic domain (Figure 5A and 5B). We found that without the extracellular and transmembrane domains, SAX-3::GFP accumulated into large aggregates in the cytoplasm of PDE neurons, which also displayed decreased SAX-3::GFP distribution in the cell membrane and axons (Figure 5D). When the extracellular domain was deleted, SAX-3::GFP accumulated in the cytoplasm but did not form large aggregates (Figure 5E). And we found that the SAX-3::GFP vesicles were distributed in the cytoplasm, cell membrane and axons. In addition, the axons were found with reduced SAX-3::GFP distribution (Figure 5E). These results suggest that the combination of the extracellular and transmembrane domains of SAX-3 are necessary for the membrane localization. As SAX-3 has an N-terminal hydrophobic signal sequence, we deleted this sequence in SAX-3 and found that that, the truncated SAX-3::GFP, which associated with vesicles, were accumulated in the cytoplasm (Figure 5F). These observations suggest that the transmembrane domain is critical for targeting SAX-3 to intracellular vesicles that may sequentially localized to cell surface and axons through the N-terminal signals by some motor proteins. Next, we examined the localization of the transmembrane domain alone, and found that it was diffusively distributed in the PDE neuron (Figure 5G). When the cytoplasmic domain was deleted, we found that, though SAX-3::GFP localized normally, the expression level was significantly decreased (Figure 5H), indicating that the cytoplasmic domain is important for SAX-3 expression level in PDE neurons. Moreover, we did not observe any GFP signal in PDE neurons when both of the cytoplasmic and transmembrane domains were removed. It is probably because the extracellular domain was secreted outside of the neurons and became invisible to microscopy. To rule out the effect of endogenous SAX-3 in the experiments, we examined the localization of full length and the truncated SAX-3 receptors in the null *sax-3(ky123)* mutant worms and the results were identical to those of in the wild-type background (Figure 5I and data not shown). All the observations above suggest that both of the N-terminal signal and the transmembrane domain of SAX-3 are necessary for its membrane localization.

## Discussion

### The Role of SAX-3 in Axon Guidance

Here we show that SAX-3 plays an important role in ventral axon guidance of PDE neurons, which is consistent with the previous report that SAX-3 is required for ventral guidance in multiple types of neurons in *C. elegans* [16]. However, dorsal axon guidance is not affected by SAX-3 as the axons of GABAergic motor neurons (visualized by GFP expression under *unc-25* promoter) are normal in *sax-3(ky123)* mutant worms (data not shown). These results suggest that SAX-3 specifically guides axons to the ventral nerve cord of *C. elegans*. SLT-1, which is the ligand for SAX-3, repels axons to promote ventral axon guidance. However, in the null *slt-1(eh15)* mutant worms, the PDE neurons displayed weak ventral axon guidance defect as compared with the PDE axon guidance defect in the *sax-3(ky123)* mutant worms (Figure 3), indicating that there are other unknown ligands for SAX-3 or that SAX-3 may function in a ligand independent manner in axon guidance [18]. The partial rescue by SAX-3::GFP or SAX-3 in the *sax-3(ky123)* mutants indicates that, although SAX-3 predominately acts in a cell-autonomous fashion, SAX-3 may also function non-cell-autonomously by other unknown mechanisms to control axon guidance of the PDE neuron. This seems different from the anterior ventral microtubule (AVM) neuron, in which SAX-3 cell-autonomously regulates ventral axon guidance [13]. The AVM neuron is born in the first larval stage, whereas the PDE neuron is born during the second larval stage. It is possible that the mechanisms for axon guidance in PDE neuron and in AVM neuron are different [37]. For example, in the *slt-1(eh15)* mutant worms, AVM neurons showed much higher percentage of axon guidance defect than PDE neurons (30% vs. 4%) [16].

### The Role of SAX-3 in Axon Outgrowth

Downstream pathways of the ligands and receptors are the small GTPases and actin-based cytoskeletal elements. It is well established that the small GTPases and the actin-associated proteins are involved in the axon outgrowth and guidance [24], [31], [38], [39]. Rac GEF and Rac GTPases work in the Robo/SAX-3 signaling to facilitate axon outgrowth in different organisms [15], [18], [40]–[42]. Here we showed that SAX-3 acted through the Rac GEF UNC-73 to stimulate axon outgrowth in PDE neurons as assayed by suppression of a gain-of-function overexpression phenotype. UNC-73 is known as a molecular switch to control the signal transduction pathway linking membrane receptors to the cytoskeleton [32], [33], [38]. It is also reported that UNC-73 could bind to the cytoplasmic domain of SAX-3. Therefore, SAX-3 may directly interact with UNC-73 to induce axon outgrowth. However, CED-10 and MIG-2, which are the two downstream effectors of UNC-73, did not participate in the SAX-3::GFP induced axon outgrowth in our experiments. UNC-73 might use other downstream factors to facilitate axon outgrowth of PDE neuron by SAX-3::GFP. This is different from MYR::UNC-40, which acted through UNC-34 and CED-10 but not UNC-73 in the AVM neuron to stimulate axon outgrowth [29]. *unc-115(ky275)*, which could suppress the MYR::UNC-40 in the AVM neuron [29], also suppressed the SAX-3::GFP in PDE neurons, indicating that both of SAX-3 receptor and UNC-40 receptor may act through the actin binding protein UNC-115 to stimulate axon outgrowth. As actin rearrangement provides structural framework and it also provides the generating force for axon initiation, axon outgrowth caused by any pathways may depend on the rearrangement of actin at the growth cone by actin associated proteins.

However, here we only described a screen that identified the potential interactors of *sax-3* in axon outgrowth of PDE neurons by exploiting an overexpressing system. Nevertheless, our work is consistent with the previous reported genetic interaction between SAX-3 and the identified genes in *C. elegans* [18], [24].

### The Membrane Localization of SAX-3

In the search for *C. elegans* mutants with abnormal SAX-3::GFP subcellular distribution, we examined the known axon guidance mutants, motor protein kinesin mutants, clathrin adapter mutants, small GTPases mutants, and autophagy signaling mutants. However, we did not identify any mutant that displayed obvious SAX-3::GFP membrane localization defect. It is reported UNC-73 and VAB-8L regulate SAX-3 in the ALM neuron [18]. The membrane localization and expression levels seemed not altered in PDE neurons of *unc-73(e936)* and *vab-8(e1017)* mutants. However, the failure to observe a gross effect of UNC-73 on SAX-3 membrane localization does not exclude the model that UNC-73 acts upstream of SAX-3 in ALM neuron [18]. The ALM neuron has a long anterior process, which is different from the ventral axon of the PDE neuron. There may exist a different mechanism regulating SAX-3 membrane localization in the PDE neurons. In the *unc-51* mutants and *unc-51RNAi* worms, we observed that a slightly higher percentage (five percent) of the PDE cell bodies containing abnormal SAX-3::GFP aggregates. It is known that early endosomal trafficking involving UNC-51 that regulates multiple types of post-Golgi vesicles to support axon growth and guidance in different types of neurons and organisms [43]. In *unc-51* mutants, several types of neurons displayed axon defects, including premature termination, abnormal trajectories, and extra axon branches [44], [45]. UNC-51 is known for its role in regulating dorsal axon guidance of motor neurons by regulating the distribution of UNC-5 [19]. In our study, SAX-3::GFP appeared to aggregate in PDE cell bodies of *unc-51* mutants and *unc-51RNAi* worms. However, the expressivity is so low, suggesting that UNC-51 may not be the only gene that regulates SAX-3 membrane localization. The membrane localization of SAX-3 is unique and may be regulated by other unidentified mechanism and genes. UNC-51 probably acts redundantly with these proteins in SAX-3 membrane localization. Further work should be performed using forward genetic screen by EMS to identify novel genes that are critical for SAX-3 membrane localization in *C. elegans.*

## Supporting Information

Figure S1 **The PDE neurons are sensitive to RNAi in the** ***rrf-3(pk1426)*** **mutants.** (A) Control RNAi treatment on *egIs1*(P*dat-1*::GFP)*;rrf-3(pk1426)* worms. (B) GFP RNAi treatment on *egIs1*(P*dat-1*::GFP)*;rrf-3(pk1426)* worms. (C) Expression levels of the autophagic genes were detected by Real-time PCR in the RNAi experiments. All real-time PCR experiments were performed three times in duplicate. Error bars represent standard errors. Asterisks denote statistically significant difference between control RNAi and specific gene RNAi. ***P<0.001.
(TIF)
