# Supplementary figures and images for: The SAX-3 Receptor Stimulates Axon Outgrowth and the Signal Sequence and Transmembrane Domain Are Critical for SAX-3 Membrane Localization in the PDE Neuron of C. elegans
Source: PLoS One. 2013 Jun 12;8(6):e65658. doi: 10.1371/journal.pone.0065658 (PMC3680500; doi:10.1371/journal.pone.0065658)

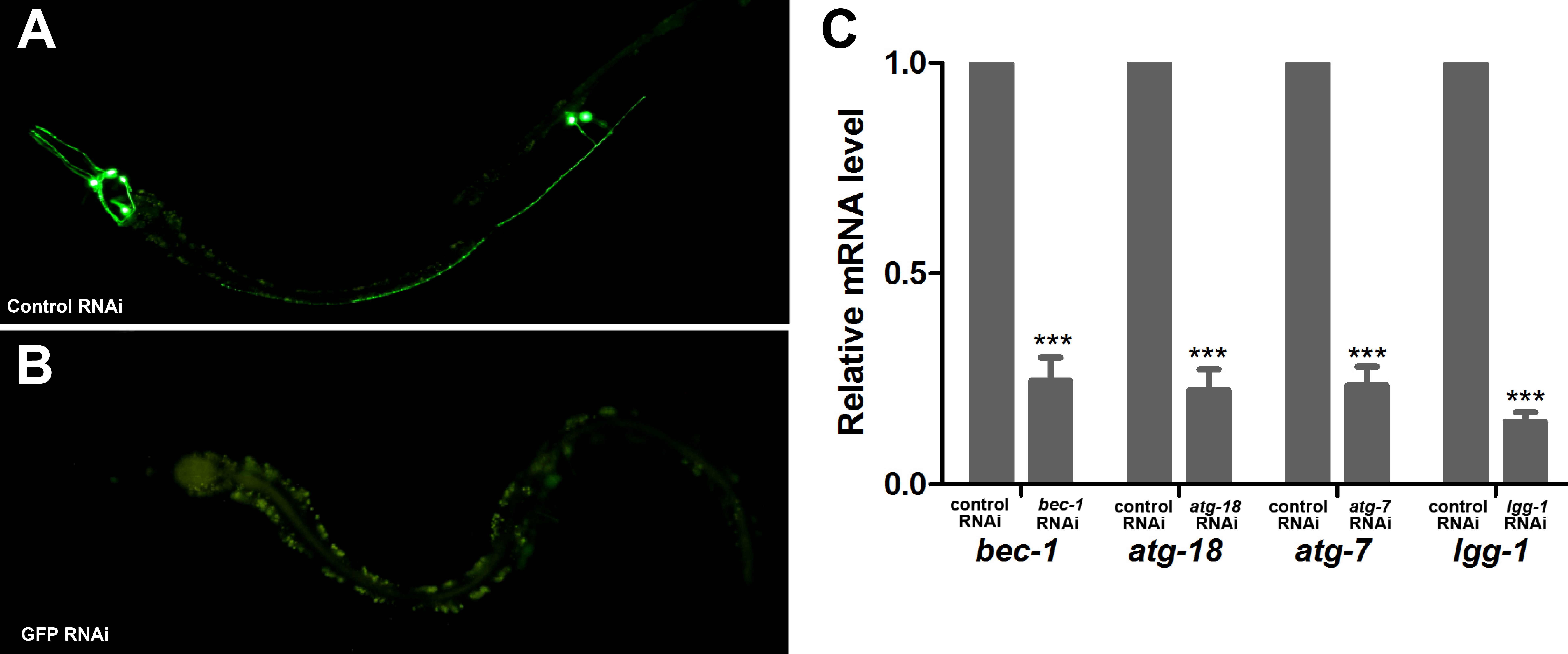

Supplement: Figure S1 — The PDE neurons are sensitive to RNAi in the rrf-3(pk1426) mutants. (A) Control RNAi treatment on egIs1(Pdat-1::GFP);rrf-3(pk1426) worms. (B) GFP RNAi treatment on egIs1(Pdat-1::GFP);rrf-3(pk1426) worms. (C) Expression levels of the autophagic genes were detected by Real-time PCR in the RNAi experiments. All real-time PCR experiments were performed three times in duplicate. Error bars represent standard errors. Asterisks denote statistically significant difference between control RNAi and specific gene RNAi. ***P<0.001. (TIF) [file pone.0065658.s001.tif]
